# Using a Designathon to Develop an HIV Self-Testing Intervention to Improve Linkage to Care Among Youths in Nigeria: Qualitative Approach Based on a Participatory Research Action Framework

**DOI:** 10.2196/38528

**Published:** 2023-06-29

**Authors:** Ifeoma Idigbe, Titilola Gbaja-Biamila, Sarah Asuquo, Ucheoma Nwaozuru, Chisom Obiezu-Umeh, Kadija M Tahlil, Adesola Zaidat Musa, David Oladele, Bill Kapogiannis, Joseph Tucker, Juliet Iwelunmor, Oliver Ezechi

**Affiliations:** 1 Nigerian Institute of Medical Research Lagos Nigeria; 2 Saint Louis University St. Louis, MO United States; 3 University of North Carolina Chapel Hill, NC United States; 4 Wake Forest Medical Center Medical Center Boulevard Winston-Salem, NC United States; 5 Saint Louis University Missouri, MO United States; 6 National Institutes of Health Bethesda, MD United States

**Keywords:** crowdsourcing, youth-led strategies, linkage to care, HIV, HIV testing, public health, participatory medicine, youth

## Abstract

**Background:**

UNAIDS (Joint United Nations Programme on HIV and AIDS) and the Nigeria National HIV/AIDS Strategic Framework recommend HIV self-testing and youth-friendly services to enhance HIV testing, linkage to health services, and prevention. However, the voices of youths are seldom incorporated into interventions. We examined qualitative data generated from a series of participatory events in partnership with Nigerian youths focused on enhancing linkage to care.

**Objective:**

The aim of this study was to assess youth-initiated interventions developed during a designathon to improve linkage to care and sexually transmitted infection services.

**Methods:**

This study conducted a designathon informed by crowdsourcing principles and the participatory research action framework. A designathon is a multistage process including an open call, a sprint event, and follow-up activities. The open call solicited Nigerian youths (14-24 years old) to develop intervention strategies for linkage to care and youth-friendly health services. A total of 79 entries were received; from this, a subset of 13 teams responded to the open call and was invited to participate in a sprint event over 72 hours. Narratives from the open-call proposals were analyzed using grounded theory to identify emergent themes focused on youth-proposed interventions for linkage to care and youth-friendly services.

**Results:**

A total of 79 entries (through the web=26; offline=53) were submitted. Women or girls submitted 40 of the 79 (51%) submissions. The average age of participants was 17 (SD 2.7) years, and 64 of 79 (81%) participants had secondary education or less. Two main themes highlighted strategies for enhancing youths’ HIV linkage to care: digital interventions and collaboration with youth influencers. A total of 76 participants suggested digital interventions that would facilitate anonymous web-based counseling, text prompt referrals, and related services. In addition, 16 participants noted that collaboration with youth influencers would be useful. This could involve working in partnership with celebrities, gatekeepers, or others who have a large youth audience to enhance the promotion of messages on HIV self-testing and linkage. The facilitators of youths’ linkage included health facility restructuring, dedicated space for youths, youth-trained staff, youth-friendly amenities, and subsidized fees. Barriers to HIV linkage to care among youths included a lack of privacy at clinics and concerns about the potential for breaching confidentiality.

**Conclusions:**

Our data suggest specific strategies that may be useful for enhancing HIV linkage to care for Nigerian youths, but further research is needed to assess the feasibility and implementation of these strategies. Designathons are an effective way to generate ideas from youths.

## Introduction

### Overview

Worldwide, approximately 1.7 million adolescents are living with HIV and 88% (1.5 million) of these adolescents reside in sub-Saharan Africa [1]. Globally, Nigeria ranks the largest country in Africa, with 3.4 million people living with HIV and an overall HIV prevalence of 1.4% [2]. This accounts for 14% of total HIV-attributable deaths [2]. The HIV prevalence in youths is estimated to be 3.5%, which is the highest among countries in West and Central Africa [3]. Nigeria is one of the few countries where mortality due to HIV in youths is still on the rise [4]. Early sexual debut, social determinants of health, and structural factors may drive HIV and sexually transmitted infections (STIs) among Nigerian youths [5]. Despite this rising risk, reports show that few youths regularly test for HIV [6,7]. To curb the spread of HIV, there is a need for a combination of interventions to increase the uptake of testing and enhance linkage to clinical services. We define linkage to care as the process of initiating and referring youths for clinical and psychosocial services, follow-up, and referrals for HIV and STIs. Linkage to care can accelerate diagnosis, increase treatment uptake, and spur healthy behaviors.

Eradicating HIV through HIV testing in Africa has been a priority by many high-income and transitional countries, although there are concerns that testing alone would not be sufficient to eradicate HIV in African countries [8]. Some believe that it is necessary to strengthen efforts to expand HIV testing and that more priorities from governments and support from global funders are needed [9,10]. Nigeria is one of the countries with poor HIV testing outcomes. To enhance linkage to care among youths, studies have been conducted to assess HIV self-testing (HIVST) among Nigerian youth. In some of these studies, it is seen that most youths support the introduction of HIVST, but education and awareness of HIVST still need to be promoted through media campaigns [9,10]. Some studies have indicated preferences for HIVST. However, these preferences appear to be influenced by several factors, including lower cost, less invasive testing method, location of testing, and linkage to care and support after testing, and promotion of self-testing among family members and the community will be beneficial for HIVST scale-up measures among young people in Nigeria [11,12]. Some studies in Nigeria have indicated that scaling up HIVST use among youths is possible to improve youth-friendly services [10-12].

UNAIDS (Joint United Nations Programme on HIV and AIDS) recommends youth-friendly services to increase linkage to care. They define youth-friendly services as clinics that provide a full range of information and services to youths in a welcoming, convenient, confidential manner and environment. The Nigerian HIV/AIDS Strategic Framework also recommends youth-friendly services to bridge the gap between testing and linkage to care [12].

In addition, Nigeria’s National HIV Strategy for youths was itself cocreated with young people, underlining the importance of youths in policy development [13]. Not many programs in Nigeria have examined youths’ involvement in cocreating HIV programs and strategies for care. There are, however, several schools of thought on how these strategies should be implemented, given the paucity of data documented about this in low- and middle-income countries (LMICs) [14]. Few studies have examined youths’ preferences related to HIV linkage to care. Many linkage interventions and services have been developed with adults in mind as the end user, ignoring the development context of youths [15,16]. This study examined qualitative data generated from a series of participatory events in partnership with Nigerian youths focused on enhancing linkage to care. This paper aims to describe the strategies focused on linking youths to care submitted by youths in the designathon open-call contest.

### Research Questions

We asked the following questions: (1) What interventions can youths participating in a designathon develop? (2) How can these interventions be used to link youths to care?

## Methods

### Study Design

**Figure 1:**
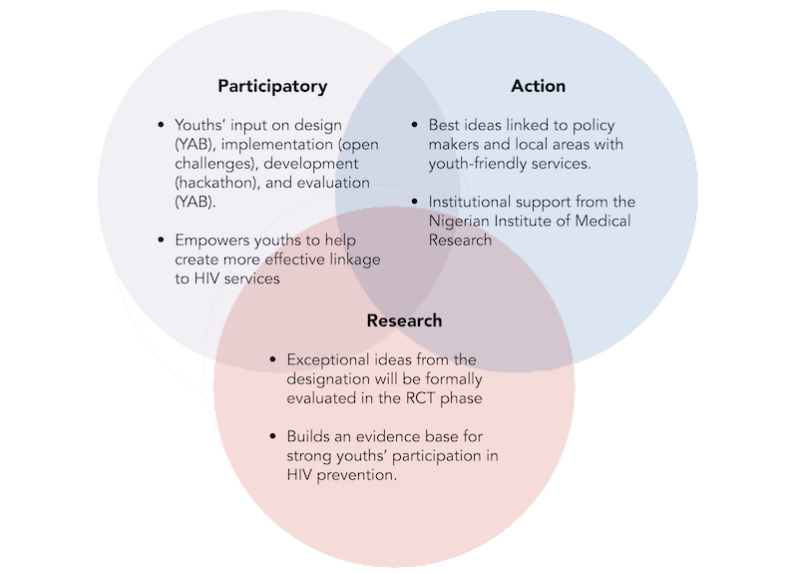
The steps taken by youths to provide innovative strategies for HIV self-testing and linkage to youth-friendly health services at a designathon. This describes the processes youths engaged in to initiate ideas and design strategies for linking youth to youth-friendly centers to access care [21]. RCT: randomized controlled trial; YAB: Youth Advisory Board.

The study used the qualitative method that focused on exploring rich data and capturing ideas and themes proposed by youths [17]. These ideas expressed in the submissions provided strategies that could be explored and implemented to effectively link youths to care. A benefit of using this method was that it created a platform for youths to express themselves, design youth-appropriate strategies, and steer the wheel [18]. Our study used a youth-led participatory action research framework. Youth-led participatory action research is an approach that engages youths to seek scientific knowledge and social change about issues related to them and advocate for change based on research evidence [19]. Employing youth-led participatory action research, youths identified a gap that in this case was linkage to care, generating data through creating ideas and developing strategies that were recommended and selected for implementation [20]. Engaging youths to brainstorm and initiate ideas that would address youth-specific situations would be an ideal process as they are suited to understand youths’ ideals and thus develop ideas and strategies to navigate the terrain [18]. In addition, using this approach created ideas tailored to youths, developed peculiar intervention strategies that were evaluated, and made recommendations to key stakeholders with the hope to implement them at the various youth-friendly facilities and institutions in the country. Involving youths in designing ideas, delivering strategies through platforms like designathons (Figure 1), and implementing will help to strengthen the vision and impact of the program in the country.

### Designathon

A designathon is a multistage crowdsourcing process including an open call, a sprint event, and follow-up activities [22,23]. We used standard methods to organize a designathon, and the purpose of the designathon was to develop strategies for linkage to care and build youths’ capacity for research [12]. We chose this format because it could bring diverse youths together, learn from peers and mentors, and help to better articulate linkage to youth-friendly services. Involving youths through this approach not only engages them but also gives them an opportunity to enjoy and own it. A total of 13 teams proceeded to a 72-hour sprint event that took place at the Nigerian Institute of Medical Research (NIMR), Lagos, between March 20, 2020, and March 22, 2020, and pitched their ideas to a judging panel. Three teams emerged as finalists from the designathon and worked with the 4 Youth by Youth team to develop their ideas (Textbox 1).

**Ideas proposed by the top 5 finalists at the designathon for linkage to care and prevention services after HIV self-testing.**

**Team 1**

Their idea entails the creation of a mobile app called iDoc that (1) addresses HIV self-testing by the watchword “doctor yourself by yourself”; iDoc chat enables the user to have a conversation with a medical doctor about confidential health issues and counseling especially on HIV/AIDS before or after conducting the test; and (2) enables users to buy an HIV self-testing kit with the app.

**Team 2**

They want to create a “safe tent” in underserved communities, where (1) young people can get comprehensive education about sexual health, HIV self-testing services, and other medical services at low cost; (2) the safe tent will be a standard structure or room, which will serve as a safe hub for the adolescents; (3) young people can receive resource materials and also partner with existing organizations, in order to achieve a productive outcome; and (4) it becomes easier for young people to access an HIV self-testing kit and health care services

**Team 3**

They propose using therapeutic communication to break the HIV self-testing and sexually transmitted infection testing barriers. They believe that therapeutic communication can be used in counseling that focuses in exploring, identifying the patient’s problems, and providing solutions to make the patient live happily.

**Team 4**

They plan to develop CRILSTA, a web- or mobile app–based digital health solution through which young people in Nigeria can have access (1) to quality information about reproductive health topics, including sexually transmitted illnesses and HIV/AIDS; (2) to a chat room where sexual health–related topics may be discussed under conditions of anonymity by users; and (3) to a mobile clinic where users can gain access to consultations with qualified medical personnel from the comfort of their homes and a health blog where information about sexual and reproductive health is shared monthly.

**Team 5**

They propose a “2-in-1” product package (OUR LEVERS): (1) lever for acceptance: general acceptance of the entry product (condoms) as earlier stated; (2) lever for publicity: social media campaigns; (3) Facebook targeted advertisements for youths aged 14-24 years; (4) Naira land ads: underneath health, romance, and family sections; (5) Twitter advertisements: geography targeting, keyword targeting, age targeting, and follower targeting for youths following online sex workers; (6) Google advertisements to drive sales; (7) lever for distribution/sensitization: sports betting shops, pharmacy stores, use of physical agents (specifically for rural dwellers), National Youth Service Corps HIV/AIDS Prevention Community Development Service groups, logistics outlets, and nongovernmental organizations such as Society for Family Health. All of these levers are interrelated and will work together for the central purpose of enabling youths to get themselves tested for HIV and make informed choices as regards their sexual and reproductive health

### Crowdsourcing Open Call

A crowdsourcing contest through an open call was organized to encourage and involve Nigerian youths (14-24 years old) to develop innovative strategies for HIVST, linkage to care (after HIVST), and youth-friendly health services. Crowdsourcing allows an intended group to solve a problem and then disseminate the solution to the public [19]. Crowdsourcing may take the form of participatory open calls to improve public health programs [22]. This open call was organized to enhance linkage to health services (after HIVST) and design youth-friendly health services. Although crowdsourcing open calls play an important part in developing ideas, they, however, do not focus on designing the final program and plan for implementation [22].

We structured our open call based on recommendations from the World Health Organization-Tropical Disease Research [24]. The open call was advertised using social media (Instagram, Facebook, and WhatsApp) and other methods (disseminating information via posters and flyers, radio jingles, online posts using peer influencers, and talking to school heads). The eligibility criteria to participate were as follows: (1) young people aged 14 to 24 years; (2) 2 to 5 members in each team submitting innovative ideas on HIVST, access, and linkage to youth-friendly centers; and (3) elicited ideas that were potentially desirable, feasible, and impactful. The participants submitted their ideas with their application using their unique team names as identifiers individually or through their schools. Their ideas were documented as words or prose, poems, pictures, or video clips. A multidisciplinary team of 8 professionals reviewed and evaluated 79 eligible submissions (26 through the web and 53 offline). Successful participants were informed and invited to pitch their ideas at the World AIDS Day contest, which took place at the Police College, Ikeja, Lagos, Nigeria, on December 1, 2019. Also, a multisectoral contest advisory committee was set up to guide the process. Finalists from the initial open call were invited to participate in the 72-hour designathon (Figure 1).

### Data Collection and Analysis

Textual data were collected using semistructured questionnaires from participants who submitted their ideas to the World AIDS Day open call. Baseline data of participants’ demographics (age, sex, the highest level of education, and gender) were collected within the submitted entries. Using the inductive approach, data were transcribed and coded using open coding to develop an initial codebook by a team (II, TG, SA, UN, COU, and KMT). The codebook included a list of question prompts, initial codes, and code meanings. Codes were reviewed, compared, analyzed, and sorted into categories to reflect consistent and overarching themes. The team developed a coding tree, deriving themes until reaching thematic saturation. We conducted descriptive statistics to characterize the demographic information of designathon participants including their age, gender, the highest level of education completed, and type of school. All analyses were performed using SPSS (version 22; IBM Corp).

### Ethics Approval

Ethics approvals were obtained from the NIMR institutional review board (IRB/18/028) and Saint Louis University (protocol number 31457). Participants were provided with detailed written forms about the study and informed of their right to decline or participate in the study. Participants who agreed to participate signed a consent form and completed the survey.

## Results

### Study Participation

A total of 79 entries (through the web=26; offline=53) were submitted from youths between the ages of 14 and 24 years. Of these entries, 33% (26/79) of the participants submitted their ideas online and 67% (53/79) submitted hard copies to a 4 Youth by Youth representative in NIMR. Most entries submitted were by youths residing in Lagos State (n=64). Approximately half of the participants were females (51%, 40/79), and more than half had at least a secondary school qualification (81%, 64/79). The average age of the participants was 17 years (SD 2.7 years); see Table 1.

We observed that themes on strategies for enhancing linkage to care focused on digital intervention and youth influencers (Textbox 2).

**Table 1:** Demographics of youths who submitted entries; Nigeria 2020 (N=79).

Demographics		Values
**Entries, n (%)**		
	Through the web	26 (33)
	Offline	53 (67)
**Gender, n (%)**		
	Female	40 (51)
	Male	39 (49)
Age (years), mean (SD)		17 (2.7)
**Geopolitical zone of residence, n (%)**		
	Southwest	61 (77)
	North-central	7 (9)
**Educational qualification, n (%)**		
	Senior Secondary Certificate Examination	64 (81)
	Tertiary	15 (19)
**Type of school, n (%)**		
	Public	62 (78)
	Private	2 (3)

**Illustrative themes that highlight ideas proposed by youths for linkage to care and prevention services after HIV self-testing.**

**Digital space**

- “Creating an interactive online customer care service where people can be educated on HIV prevention and HIV self-test” (#66 through the web)
- “iDoc chat enables the user to have a conversation with a medical doctor about confidential health issues and counselling especially on HIV/AIDs before or after conducting the test” (#16 through the web)

**Gadgets**

- “Inventing fingerprint phones, which can use fingerprint, scanning for STI” (#106 offline)
- “Developing a common chip carrying bracelet for HIV testing and linking service” (#79 offline)

**Models or gatekeepers**

- “We will engage volunteers, who are willing to work with young people on a regular basis, and they will be trained to effectively manage the safe hub” (#42 through the web)

**Competitions**

- “Naira Marley has a followership and he could be used as an ambassador of a challenge like Marley challenge.” The Marley Challenge is a challenge that is used to tell people to know their status (#84 offline)

### Digital Intervention

In total, 76 youths from the open call advocated for digital interventions to get youths in Nigeria to be linked to care and access STI services after taking a self-test for HIV. A participant stated: “A lot of youth we will be providing services to you are on their phones on social media platforms” (#58)*.* This strategy was proposed because they believed that a large proportion of youths are tech-savvy and predisposed to exploring and seeking information, education, and entertainment through digital routes. Most participants (n=76) suggested using mobile and smartphone apps as an innovative strategy to get youths to link to care and access services. Digital interventions have been known and documented to improve access to services through social media platforms and various apps, although not all youths will have smartphones of good bandwidth; they explore alternative options such as using Wi-Fi to browse and have access to social media platforms [25]. Furthermore, Unstructured Supplementary Service Data (USSD) and SMS text messaging prompts and reminders could link youths who own basic cell phones to care. USSD is a technology platform where information is transmitted on a Global System through Mobile Communication network to a basic phone. USSD and frequent SMS text messaging reminders can be used for youths who only have cell phones but not smartphones.

### Influencers

An influencer is someone who has a large following and can influence his or her followers and potential buyers of products or services by endorsing or promoting the items on social media [26]. Approximately 16 youths proposed using influencers who have a large youth fan base as a strategy to increase the uptake of HIVST and access to linkage to care. A participant stated: “Influencers and celebrities have lots of youth who follow and listen to them so this will be a good way to get youth to participate” (#32).

### Facilitators to Linkage to Health Services

Facilitators highlighted by youths that could promote or inhibit them from being linked to care included health facility restructuring, health provider engagement, aesthetic, and youth-friendly amenities (dedicated and confidential centers for youths, youth-trained staff, and low and affordable service fees for management and care).

#### Health Facility Restructuring

Many participants (n=36) believed that restructuring health facilities to have greater appeal among youths will enhance linkage to health services. Many clinical services have an adult default and do not respond to the preferences, styles, and culture of youths. Also, 23 youths stated that it is paramount to include dedicated centers in health facilities that will address the specific needs of youths and provide care and STI services for them.

#### Health Provider Engagement Youth-Friendly Services

Some participants (n=20) are of the opinion that youths including themselves will be happy to go to health centers that have youth-friendly staff. Youths are more likely to access services and adhere to procedures if there are staff they can bond with and who are trained to identify, respect, manage, and resolve youth-specific needs. Also, 12 respondents stated that youths will migrate toward centers that offer youth-specific activities. Therefore, having youth centers that have activities that will interest youths such as social-educational clubs, recreation, and health talks focused on youths will be a good avenue to embed care and STI services.

#### Aesthetics and Amenities

Few participants (n=8) assert that youths will be attracted to youth-friendly centers that are stylish, funky, and have verve and aesthetics. They can identify with pictures of hip-hop artists and celebrities. Furthermore, colorful wall paintings and furniture, installing mini arcade sections, and board games can attract the youths and make them feel comfortable in the environment and create trust in accessing the health service.

### Barriers to the Uptake of Youth-Friendly Services

Youths may not access care based on the following factors:

- Lack of discrete signage: participants (n=12) suggest that youth-friendly centers or health facilities that provide STI services for youths should have discrete signs to make youths feel comfortable about accessing such services. However, there are signposts that highlight activities that are indicative of sexuality, deviant behavior attributes, and promiscuity in extreme cases. This can be frowned at or misconstrued based on the cultural beliefs and perceptions in the Nigerian setting irrespective of the intended positive outcome, which is to encourage youths to get tested for HIV and other STIs.
- Lack of privacy and confidentiality: participants (n=7) state that they will be discouraged from getting linked to care or accessing STI services if they do not feel comfortable or are referred to health centers that do not offer some level of privacy during counseling and testing or if the information provided by them is not kept confidential.

### Poor Communication and Neglecting Youths’ Ideas

In total, 39 youths believe that if stakeholders do not involve youths in planning, decision-making, and implementation, and incorporate their ideas and strategies in the various health facilities, the uptake of these services by youths will be poor. Also, communication gaps are created if ideas, suggestions, and feedback are provided in thematic areas when youths converge at various social institutions but are not channeled to the appropriate stakeholders responsible for youths’ development in the country.

## Discussion

### Principal Findings

Our study examined qualitative data generated from a series of participatory events in partnership with Nigerian youths focused on strategies for enhancing linkage to care (Textbox 1). We assessed youth-initiated interventions developed during a designathon to improve linkage to care and STI services, and the aim was to invite youths through an open call to a 3-day designathon where they developed strategies and pitched their ideas on how youths could be engaged and linked to care and STI services. Furthermore, ideas from the finalists were recommended and implemented at selected youth-friendly facilities. This study extends the literature by focusing on a designathon in an LMIC setting, capturing data from diverse youths’ voices, and creating strategies to increase linkage to access to care. Nigerian youths propose that imploring digital interventions and engaging youth influencers will be effective strategies to link them to care.

Many youths proposed digital interventions to enhance linkage to care. This finding is like qualitative literature supporting digital interventions for linkage to HIV services [27]. Digital interventions can reach, engage, and retain youths and young adults in HIV prevention and care interventions [23,28,29]. Reports show that 70% of young Nigerians access social media platforms [23], underlining the potential promise of digital interventions in this context. At the same time, reaching youths without cell phones is also an important consideration.

Our data suggest that collaborating with youth influencers may enhance linkage to care. This is consistent with a larger literature that showed that influencers engaged in sexual health communications could effectively create awareness and disseminate information [29]. Influencers may have a better understanding of youths’ preferences, opinions, experiences, and needs. This could translate into benefits for promoting HIVST and linking to care. Involving influencers who endorse the program could provide more visibility. Incorporating these strategies and interventions in HIV programs at the different structural levels could help increase the uptake of HIV services among youths in Nigeria [12,29-31]. At the same time, this strategy carries risks such as trying to break the culture and mindset in LMICs where youths’ opinions may not be taken seriously to the ways in which youths may tend to express their opinions, which may not align with practices in LMICs [32]. Thus, further implementation research for incorporating youth-driven programs is needed [32].

Our data suggested several facilitators and barriers to accessing youth-friendly services. This is like a study that examined peer-led interventions to reduce HIV risk in LMICs [32]. However, there is limited analysis of facilitators and barriers considering the diverse sociocultural settings that vary for youths in Nigeria. There is a need to mitigate these barriers and further explore and test the facilitators on how they could influence and maximize youth’s linkage to care.

Involving youths in idea implementation and positioning them as stakeholders while executing the ideas as shown in Textbox 1 provides leverage, independence, and confidence for them as social actors to determine proactive strategies that work for them and have an impact value as the strategies were created by them, and positive outcomes. This can be achieved from programs focused on health research for youths in the country such as this designathon. It also created an opportunity for youths to come from different parts of the country, thus creating proper representation to interact, collaborate, fuse, and pitch their innovative ideas, which could be piloted in certain social institutions within the country. Engaging, supporting, and working with youths in the design of HIV prevention approaches can create strategies that influence and possibly promote uptake of HIVST among youths to bring about the desired action, outcome, and attitude toward linkage to care and prevention services in various youth-friendly structural settings or institutions in the country.

### Strengths and Limitation

Our study has several important limitations. Our themes were identified from a sample of Nigerian youths who responded to an open call, and this group does not represent all Nigerian youths. Second, the interventions proposed were based on ideas from youths within specific geopolitical zones, which may not be applicable in other geopolitical zones because of the multicultural characteristics and heterogeneous nature of the country. Third, few designathon follow-up activities have been conducted. Further research is needed to understand the effectiveness of implementing ideas generated during the designathon. The observed strength of this study was the use of crowdsourcing. Using crowdsourcing encouraged a bottom-up approach with the youths, which gave all the participants equal opportunity to be part of providing a solution to linkage to care. Crowdsourcing in this study provided enhanced innovative ideas, better participant engagement, greater diversity of thinking, and a rich source of participant data.

### Conclusions

Youths should serve as stakeholders and be involved in participatory events in activities that focus on youths to optimize the quality of care and services. Designathons have shown to be an interactive, engaging, and effective means of creating ideas and strategies for health promotion in an LMIC setting. Collaborating with and including youths as stakeholders in participatory events initiated and geared toward improving their health outcomes provide them the opportunity to create ideas and develop strategies for evaluation and implementation. Thus, taking the required steps in health promotion, adding to existing knowledge, providing representation from the Nigerian standpoint, and contributing its quota toward achieving the overarching goal will help reduce the burden of HIV among youths in Nigeria.

## Abbreviations

HIVST: HIV self-testing
LMIC: low- and middle-income country
NIMR: Nigerian Institute of Medical Research
STI: sexually transmitted infection
UNAIDS: Joint United Nations Programme on HIV and AIDS
USSD: Unstructured Supplementary Service Data
